# Soil Fauna Alter the Relationship Between Plant Litter Diversity and Microbial Communities in Mixed Litter Decomposition

**DOI:** 10.1002/ece3.72992

**Published:** 2026-01-30

**Authors:** Pengpeng Dou, Dunmei Lin

**Affiliations:** ^1^ Institute of Ecological Conservation and Restoration Chinese Academy of Forestry Beijing China; ^2^ Institute of Desertification Studies Chinese Academy of Forestry Beijing China; ^3^ Key Laboratory of the Three Gorges Reservoir Region's Eco‐Environment, Ministry of Education Chongqing University Chongqing China

**Keywords:** functional diversity, litter decomposition, litter traits, phospholipid fatty acid, soil fauna

## Abstract

Understanding how litter diversity and soil fauna drive microbial communities is critical for revealing trophic cascades in decomposition processes. We conducted a 460‐day field decomposition experiment in a subtropical forest, placing litter mixtures of one to four tree species into mesh bags with 25 μm or 4 mm openings to create fauna‐excluded and fauna‐accessible treatments, and assessing microbial community composition and biomass using phospholipid fatty acid (PLFA) analysis. The results showed that the litter traits and diversity significantly influenced microbial PLFA content. Both fungal and bacterial PLFA content increased with higher levels of nitrogen, phosphorus, manganese, potassium, and leaf thickness in the mixed litter. Soil fauna had a significant impact on microbial PLFA content and its community distribution. A notable interaction between soil fauna and litter richness was observed: soil fauna slowed the decline of bacterial PLFA content as litter richness increased, highlighting their role in moderating the impact of litter diversity on microbial communities. Additionally, soil fauna reversed the relationship between mixed litter traits and arbuscular mycorrhizal fungi (AMF) PLFA content. While a negative correlation was observed in the absence of soil fauna, their presence turned it positive, demonstrating that soil fauna modifies the impact of litter traits on AMF. These findings demonstrate the intricate interactions between plant litter diversity, soil fauna, and microbes, highlighting the crucial role of soil fauna in modulating the effect of plant diversity on microbial communities during decomposition.

## Graphical abstract

This study reveals a complex interplay among litter diversity, soil fauna, and microbial communities in subtropical forests. Soil fauna not only significantly affect microbial biomass and community composition, but also mitigate the negative effects of plant diversity on bacterial communities and reverse the associations between specific microbes and litter traits. These results emphasize the pivotal bridging role of soil fauna in regulating plant diversity–microbial relationships during decomposition and offer new insights into trophic‐cascade dynamics.

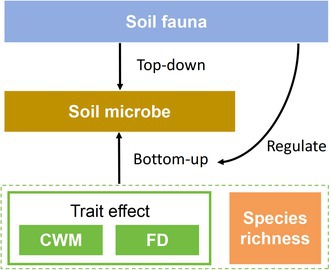

## 1 Introduction

Litter decomposition plays an important role in carbon (C) and nutrient cycling in ecosystems (Handa et al. 2014; Xu et al. 2020). More than 90% of net primary production enters the soil through plant litter, thus supporting complex food webs (Gessner et al. 2010). During litter decomposition, the activities of colonizing microorganisms, in addition to physical leaching, are the main biological mechanisms driving decomposition. Studies have shown that the trait effects of mixed litter (Sena et al. 2018; Wu et al. 2023), litter richness (Jonsson and Wardle 2008), and soil fauna involvement (Njoroge et al. 2023; Wu et al. 2014) can influence microbial communities through “bottom‐up” (resource regulation) or “top‐down” (predation regulation) pathways, thereby regulating microbial community composition and function. However, global biodiversity loss is seriously threatening the stability of ecosystem functioning (Hooper et al. 2012; Mori et al. 2020), with microbial‐mediated decomposition cascades as key agents of particular vulnerability.

In the bottom‐up model, plant litter physicochemical traits are closely related to microbial activity and decomposition efficiency (Gillespie et al. 2021; Ridgeway et al. 2022). As a food source, litter can directly influence microbial activity. Specifically, the nutrient content and physical properties of mixed litter, such as nitrogen and carbon content, can regulate microbial metabolism and influence their phospholipid fatty acids (PLFA) profiles (Zheng et al. 2018). Also, litter affects the formation of decomposing microhabitats on the forest surface (Perez‐Harguindeguy et al. 2000), helping microorganisms cope with environmental changes (Malik et al. 2020). For example, removing understory litter significantly reduced fungal PLFA levels, whereas litter mixing notably enhanced the abundance and diversity of microbial communities on leaves (Liu et al. 2022; Wan et al. 2021). Based on the mass ratio hypothesis, the relevance of litter traits is more closely tied to the traits of dominant species than to species diversity, and mixed litter traits can be represented by the community‐weighted mean (CWM) trait values of the constituent species (Tardif and Shipley 2015). Studies have shown that initial litter traits (e.g., C/N ratios) are key factors that dominate microbial decomposers in subtropical broadleaf evergreen forests (Li et al. 2020). Furthermore, mixtures containing the species with the greatest differences in initial nitrogen content exhibited greater synergistic effects (Cuchietti et al. 2014). Such differences in functional traits, rather than simply litter richness, play a key role in promoting complementarity within mixed litter. In particular, functional trait variation among species is especially important in communities with high litter richness (Hattenschwiler et al. 2005), and this variation can significantly influence microbial PLFA composition and diversity. Significant differences in the composition and biomass of microbial species were observed with changes in the tree species richness (Pei et al. 2017; Zhang et al. 2018). However, plant species richness did not affect the decomposition of recalcitrant materials (Cong et al. 2015). This may indicate that while plant species richness promotes microbial diversity, it might not directly influence microbial PLFA in the decomposition of more resistant materials. Thus, the relationship between mixed litter traits, litter richness, and microbial PLFA remains unclear, particularly within the same experimental framework, highlighting the need for further research.

**FIGURE 1:**
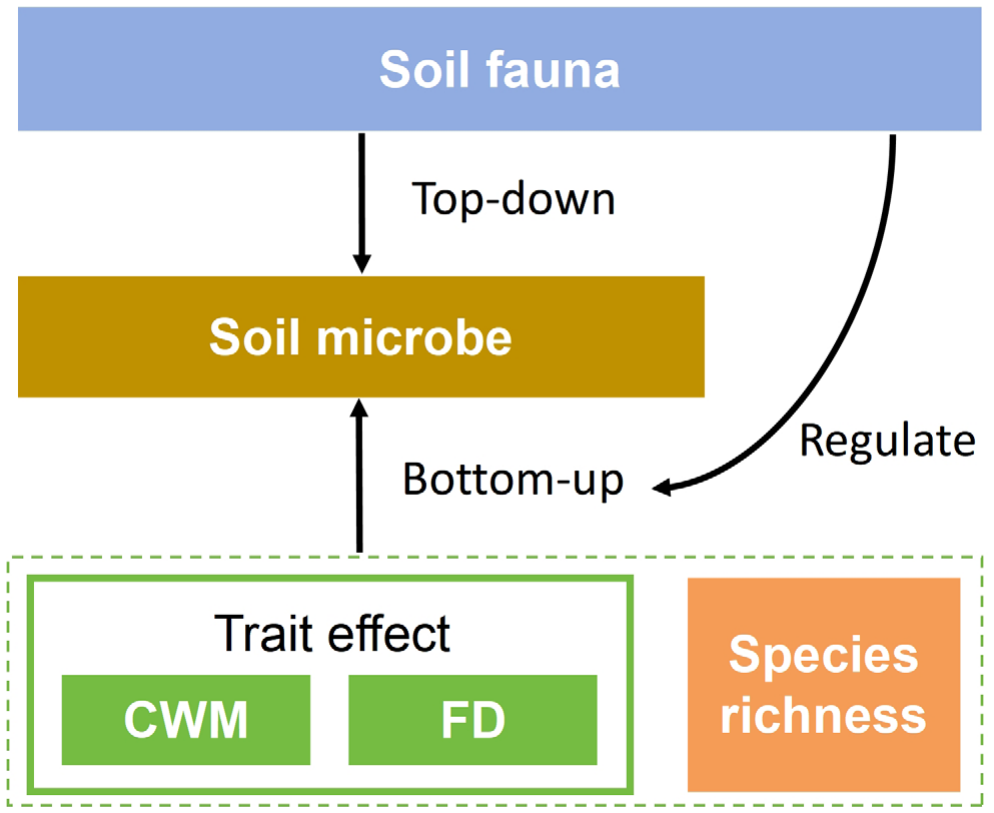
A concept map illustrates the three‐level interactions between litter characteristics, soil fauna, and microorganisms.

In the top‐down model, microorganisms showed selective utilization of leaf litter (De Oliveira et al. 2010; Vos et al. 2011). For instance, bacteria preferentially utilize high‐quality litter with higher nitrogen content, while fungi are dominant in decomposing more recalcitrant litter (Yang et al. 2021). Simultaneously, microbial diversity increases with the rise in trophic levels within the soil food web (Zhu et al. 2021). On one hand, soil fauna can increase the surface area available for microbial colonization through litter fragmentation (Njoroge et al. 2022). Soil fauna, such as earthworms, collembola, nematodes, and microfauna, can also influence microbial community composition by selectively grazing different microbial populations, which in turn affects microbial PLFA profiles (Kane et al. 2023; Wee et al. 2024; Yang et al. 2024). On the other hand, different types of soil fauna have diverse and unique microbial communities through selective grazing (Potapov et al. 2019; Zhu et al. 2021). For instance, some key fungal taxa have an advantage in degrading recalcitrant plant materials, thereby supporting the growth of other bacterial and fungal taxa by releasing easily degradable compounds (Habtewold et al. 2020). However, the presence of macrofauna can also slow the decomposition of recalcitrant substances like phenolics and tannins (Ristok et al. 2019). Thus, the influence of soil fauna on microbial PLFA profiles may be related to the type and abundance of fauna present, highlighting their significant role in shaping microbial communities through both direct and indirect interactions (Figure 1).

Understanding the biological interactions involving soil fauna across different nutrient levels is essential to comprehending microbial community dynamics. Soil fauna influence microbial communities not only through direct effects such as grazing and litter fragmentation but also by altering the physical and chemical properties of the litter itself. For instance, soil fauna may modify litter decomposition rates, nutrient availability, and litter structure (Joly et al. 2018), which can change the relationship between litter traits and microbial PLFA. Soil fauna may alter nutrient release from decomposing litter (Peguero et al. 2019), particularly in nutrient‐poor sites, which subsequently affects microbial community structure and PLFA composition. Additionally, soil fauna can modify the microhabitats within the litter layer (Peng et al. 2023), affecting microbial colonization patterns and the competitive interactions between different microbial groups. This, in turn, shapes the diversity and functional composition of microbial communities. Nonetheless, few studies have simultaneously considered the interactions between plants, soil fauna, and microorganisms during the decomposition of mixed litter.

To examine the complex trophic cascade effects during litter decomposition, we conducted a 460‐day field experiment using mixed litter consisting of one to four species and litterbags with two mesh sizes. This study aims to systematically investigate the interactions across trophic levels and their effects on microbial communities under the same experimental conditions, with a particular focus on the direct and indirect regulatory roles of soil fauna. Specifically, we aim to assess how the richness and functional traits of mixed plant litter influence the composition of microbial communities and their PLFA profiles. Additionally, we will explore how soil fauna modulate these effects. We hypothesized that: (1) Mixed litter traits, along with their diversity and litter richness, will influence microbial PLFA. The chemical and physical properties of mixed litter (such as nitrogen content and C/N ratio) directly affect microbial community composition and metabolism by regulating nutrient availability. The diversity and richness of litter provide a variety of resources and ecological niches, promoting microbial diversity and resulting in a more diverse microbial PLFA. (2) Soil fauna will significantly impact microbial community structure and PLFA. Soil fauna, through selective grazing, would drive shifts in microbial community composition by altering the dominant microbial groups, thereby affecting the PLFA and (3) Soil fauna will modify the relationship between litter traits and microbial PLFA. Soil fauna, by fragmenting litter and facilitating nutrient release, will directly or indirectly alter microbial habitat availability and nutrient dynamics, influencing microbial community composition, which will be reflected in changes in microbial PLFA.

## 2 Materials and Methods

### 2.1 Study Area

This study was conducted in a subtropical evergreen broad‐leaved forest located in the Jinyunshan National Nature Reserve in Chongqing, southwestern China (29°41′–29°52′ N, 106°17′–106°24′ E). The site experiences a subtropical monsoon climate, averaging 13.6°C in temperature and 1612 mm in annual rainfall (Yu et al. 2015). Soils are acidic yellow soils (Song et al. 2018). The forest canopy is dominated by evergreen species such as *Symplocos lucida* Thunb., Siebold & Zucc., *Castanopsis fargesii* Franch, *Machilus nanmu* (Oliv.) Hemsl., and *Elaeocarpus japonicus* Siebold & Zucc.

### 2.2 Litter Decomposition Experiment

Freshly senesced leaf litter from four dominant tree species—*Castanopsis fargesii* (C), *Elaeocarpus japonicus* (E), *Machilus nanmu* (M), and *Symplocos lucida* (S)—was collected for this study. Sampling was conducted in spring 2016, with litter obtained from at least 10 mature individuals per species. To ensure quality, we excluded leaves showing evidence of herbivory damage or fungal infection. For a detailed description of the processing methods, please refer to Dou and Lin (2025b).

In this study, we used two types of litterbags with different mesh sizes. 25 μm mesh size litterbags were used to allow microbes access but exclude most of the soil fauna, while litterbags with 4 mm (upper part of the litter bags) and 0.5 mm (lower part facing the soil surface) were used to allow access of the soil fauna while avoiding loss of litter fragments. Each litterbag (10 cm × 15 cm in size) was filled with 3 g litters of different species combinations as follows: litter bags contained single species (3 g each of C, E, M, and S), two‐species mixtures (1.5 g of each species in all possible combinations), three‐species mixtures (1 g of each species in all possible combinations), and a four‐species mixture (0.75 g of each species), resulting in 15 distinct litter treatments. The litterbags were placed in four blocks. In each block, there were 30 litterbags, corresponding to 15 treatment types × 2 litterbag types, which were randomly distributed. The litterbags were fastened to the forest floor using plastic nails. After 460 days of field exposure, all litterbags were harvested, transferred to plastic bags, and stored at −80°C for microbial community analysis.

### 2.3 Characterization of Litter Traits

Initial traits of the litter, including physical traits [leaf thickness, toughness, standard water‐holding capacity (SWHC), and specific leaf area (SLA)] and chemical traits [concentrations of C, N, P, potassium (K), and manganese (Mn), and lignin content] were measured. Leaf thickness, toughness, and SLA were measured using 5 individual litter samples, while SWHC was assessed with 10 samples, SWHC with 10 samples, and chemical traits with three samples. We measured leaf thickness with a thickness meter, while toughness was assessed as the tearing resistance (force per unit width) using a tensile testing apparatus (Laird‐Hopkins et al. 2017). Leaf SWHC is the percentage of water absorption of the water‐soaked litter for 1 h (Dou et al. 2018). SLA is the ratio of leaf litter area to its dry mass (Vile et al. 2005). Leaf area was measured using a scanner. Dry litter mass was weighed after being oven‐dried at 60°C for 48 h.

C was measured directly in a Shimadzu TOC/TN analyzer with the SSM‐5000 model. After digestion with 98% H_2_SO_4_ and 30% H_2_O_2_, N of the litter was measured on the Shimadzu TOC/TN analyzer. P was measured through the Mo‐Sb anti‐spectrophotometer method. The concentrations of K and Mn were measured using an inductively coupled plasma emission spectrometer. The lignin content was measured through the acid washing method. We also calculated C/N, C/P, and N/P for each litter species (Table 1).

**TABLE 1:** Initial physical and chemical properties of leaf litter.

Species combination	C content (mg/g)	N content (mg/g)	P content (mg/g)	C/N	C/P	N/P	K content (mg/g)	Mn content (mg/g)	Lignin content (mg/g)	Thickness (μm)	Toughness (N/mm)	SLA (m^2^/kg)	SWHC (%)	FDis
C	477.85^ij^	11.69^b^	0.59^h^	41.32^a^	811.08^ab^	19.88^ab^	3.51^h^	2.22^gh^	134.94^a^	287.4^a^	0.62^acd^	8.82^a^	28.59^fg^	
E	443.02^ef^	6.57^a^	0.17^a^	68.48^a^	2669.06^h^	39.94^c^	2.69^bc^	1.79^e^	185.73^e^	249.4^a^	0.39^a^	10.13^a^	23.2^bf^	
M	492.73^k^	6.97^a^	0.46^dfg^	73.82^a^	1079.44^bc^	15^a^	2.16^a^	0.83^a^	344.13^j^	257.4^a^	0.65^bcd^	9.98^a^	30.75^g^	
S	380.96^a^	8.68^ab^	0.53^fh^	44.91^a^	737.75^a^	16.85^ab^	4.26^j^	2.26^h^	148.98^bc^	312^a^	0.93^e^	8.74^a^	10.67^a^	
CE	460.43^g^	9.13^ab^	0.38^bcd^	54.9^a^	1740.07^fg^	29.91^bc^	3.1^ef^	2.01^f^	160.34^d^	268.4^a^	0.5^ab^	9.47^a^	25.89^cdfg^	2.66
CM	485.29^jk^	9.33^ab^	0.53^fh^	57.57^a^	945.26^ab^	17.44^ab^	2.84^cde^	1.53^c^	239.53^h^	272.4^a^	0.64^acd^	9.4^a^	29.67^fg^	2.44
CS	429.4^c^	10.18^ab^	0.56^gh^	43.11^a^	774.41^a^	18.37^ab^	3.89^i^	2.24^gh^	141.96^ab^	299.7^a^	0.78^ce^	8.78^a^	19.63^bc^	1.81
EM	467.88^gh^	6.77^a^	0.31^b^	71.15^a^	1874.25^g^	27.47^ac^	2.43^ab^	1.31^b^	264.93^i^	253.4^a^	0.52^ac^	10.06^a^	26.97^dfg^	2.16
ES	411.99^b^	7.62^ab^	0.35^bc^	56.69^a^	1703.4^eg^	28.39^ac^	3.48^gh^	2.03^f^	167.35^d^	280.7^a^	0.66^bcd^	9.44^a^	16.94^ab^	3.01
MS	436.84^ce^	7.82^ab^	0.49^efh^	59.36^a^	908.59^ab^	15.93^ab^	3.21^fg^	1.55^c^	246.55^h^	284.7^a^	0.79^de^	9.36^a^	20.71^bd^	2.99
CEM	471.2^hi^	8.41^ab^	0.41^bcde^	61.21^a^	1519.86^def^	24.94^ab^	2.79^cd^	1.62^c^	221.6^g^	264.73^a^	0.55^acd^	9.64^a^	27.51^efg^	2.79
CES	433.94^cd^	8.98^ab^	0.43^cf^	51.57^a^	1405.96^de^	25.56^ac^	3.49^gh^	2.09^fg^	156.55^cd^	282.93^a^	0.65^acd^	9.23^a^	20.82^bd^	2.89
CMS	450.51^f^	9.11^ab^	0.53^fh^	53.35^a^	876.09^ab^	17.25^ab^	3.31^fh^	1.77^de^	209.35^f^	285.6^a^	0.73^bce^	9.18^a^	23.34^bf^	2.78
EMS	438.9^de^	7.41^a^	0.38^bcd^	62.4^a^	1495.41^def^	23.93^ab^	3.04^df^	1.63^cd^	226.28^g^	272.93^a^	0.66^bcd^	9.62^a^	21.54^bde^	3.15
CEMS	448.64^f^	8.48^ab^	0.44^cf^	57.13^a^	1324.33^cd^	22.92^ab^	3.16^f^	1.78^de^	203.44^f^	276.55^a^	0.65^bcd^	9.42^a^	23.3^bf^	3.10
CV(%)	6.51	16.14	25.22	16.87	40.30	29.54	17.20	21.95	27.78	6.11	20.25	4.62	22.69	15.49

*Note:* Different letters indicate significant differences (*p* < 0.05). Data are presented as mean (chemical traits, *n* = 3; Thickness, toughness, and SLA, *n* = 5; SWHC, *n* = 10).

Abbreviations: C, *Castanopsis fargesii*; CV, coefficient of variation; E, *Elaeocarpus japonicus*; FDis, functional dispersion; M, *Machilus nanmu*; S, *Symplocos lucida*. SLA, specific leaf area; SWHC, standard water‐holding capacity.

To explore the effects of traits (functional identity, FI, or functional diversity, FD) of mixed litter on decomposition, we calculated the value of the trait of the community‐weighted mean (CWM) and functional dispersion (FDis; Lavorel et al. 2008).

(1) $$\mathrm{CWM}=\sum p_{i}\times {\text{trait}}_{i}$$

where $p_{i}$ was the proportion of species *i* in the mixture, and ${\text{trait}}_{i}$ was the trait value of species *i*. We used a PCA for the CWM value of the 13 traits to avoid co–linearity among the CWM trait variables. Trait values were first standardized using the method of *z* scores (mean = 0, variance = 1). CWM1 and CWM2 (as the first and second components of the PCA, respectively) would be two independent composite variables representing FI of mixed treatments. To calculate FDis, we used the ‘FD’ R package described by Laliberte and Legendre (2010).

### 2.4 Characterization of the Microbial Community

The microbial biomass and the composition of the community were estimated by extracting phospholipid fatty acids (PLFAs) from freeze‐dried litter samples using the method described by Bossio and Scow (1998). The concentration of each PLFA was determined by MIDI software (MIDI Inc., Newark, DE) using gas chromatography (Hewlett‐Packard 6890 series GC, FID) and calculated by 19:0 internal standard concentrations. A total of 27 PLFAs were used to indicate the composition of the microbial community and to estimate bacterial, fungal, and total microbial PLFAs. The PLFA of Gram‐positive bacteria (GP) was quantified as the sum of a15:0, i15:0, i16:0, a17:0, i17:0, i14:0, a12:0, a13:0, i13:0, a14:0, a16:0 (Kourtev et al. 2002), while Gram‐negative bacteria (GN) were quantified as the sum of 10:0 3OH, 12:0 2OH, 16:1 ω7c, 17:0 cy ω7c, 18:1 ω5c, 18:1 ω7c, 18:1 ω7c 10me, 19:0 cy ω7c, 12:00, 14:00, 15:00, and 17:00 (Kourtev et al. 2002). Fungal PLFA was calculated as the sum of 16:1 ω5c, 18:1 ω9c, 18:2 ω6c, and 18:3 ω6c (Kourtev et al. 2002). Specifically, 16:1 ω5c represented arbuscular mycorrhizal fungi (AMF) (Madan et al. 2002), while 18:1 ω9c and 18:2 ω6c represented saprotrophic and ectomycorrhizal fungi. Furthermore, we calculated the GP to GN ratio and the fungi to bacteria ratio.

### 2.5 Statistical Analyses

First, two‐way ANOVA was used to explore the influence of species combination, litterbag types, and their interaction on microbial community (such as AMF, fungi, and bacteria) and its structure (the ratio of fungi to bacteria and GP to GN, respectively). To ensure the data meet the assumptions of normality and homogeneity of variance, necessary transformations were applied. Specifically, the arbuscular mycorrhizal fungi PLFA data were square root (sqrt) transformed, while all other data, except for the “Gram‐positive/Gram‐negative bacteria” data, were log‐transformed. Then *t*‐tests were used to test the PLFA difference between fungi and bacteria, and GP and GN. Second, linear mixed‐effects models (LMMs) were applied to evaluate the effects of litter trait (CWM1 and CWM2), trait diversity (FD), litterbag types (soil fauna involvement or not), and their interactions (as fixed effects) on microbial PLFA, with block, litter combination, and richness incorporated into the models as random effects. We also used LMMs to assess the effects of litter richness, litterbag types, and their interactions (as fixed effects) on microbial PLFA, with block and litter combination incorporated into the models as random effects. Third, Nonmetric multidimensional scaling (NMDS) was used to analyze the distribution of microorganisms in different litterbag types. Permutation multivariate ANOVA was used to evaluate the effects of litter richness and litter combination on microbial biomass within different litterbag types. All analyses were performed in R version 3.4.2, and *p* < 0.05 was considered statistically significant.

## 3 Results

### 3.1 Litter Initial Traits in Individual and Mixed

The initial traits of the 15 different leaf litter treatments exhibited significant variation, except for C/N, thickness, and SLA (Table 1). The coefficient of variation (CV) for C/P was the highest at 40.30%, with *Elaeocarpus japonicus* (2669.06) having a value three times higher than *Symplocos lucida* (737.75). The CV of SLA was the smallest, with the CV of other traits ranging from 6.11% to 29.54%. *Symplocos lucida* had the lowest C content, which was significantly different from other treatments. However, the K and Mn content and the toughness value of *Symplocos lucida* were the highest. *Castanopsis fargesii* had a high content of N and P, but its lignin content was significantly lower. *Elaeocarpus japonicus* had the highest C/P and N/P ratios. *Machilus nanmu* had the largest SWHC, but its K and Mn content were significantly lower.

The principal component analysis of the initial weighted trait (CWM) values for the 11 litter combinations showed that the first axis (CWM1) and the second axis (CWM2) accounted for 61.22% and 24.81% of the total variation, respectively (Figure S1). The CWM1 score was negatively correlated with SLA, C/N, C/P, lignin content, SWHC, C, and N/P, and positively correlated with leaf thickness, K content, toughness, Mn, P, and N content. The score of CWM2 was negatively correlated with P content, lignin content, C content, SWHC, toughness, N content, C/N, and thickness, and positively correlated with N/P, C/P, Mn, K content, and SLA.

### 3.2 Microbial Community Composition and Biomass

**FIGURE 2:**
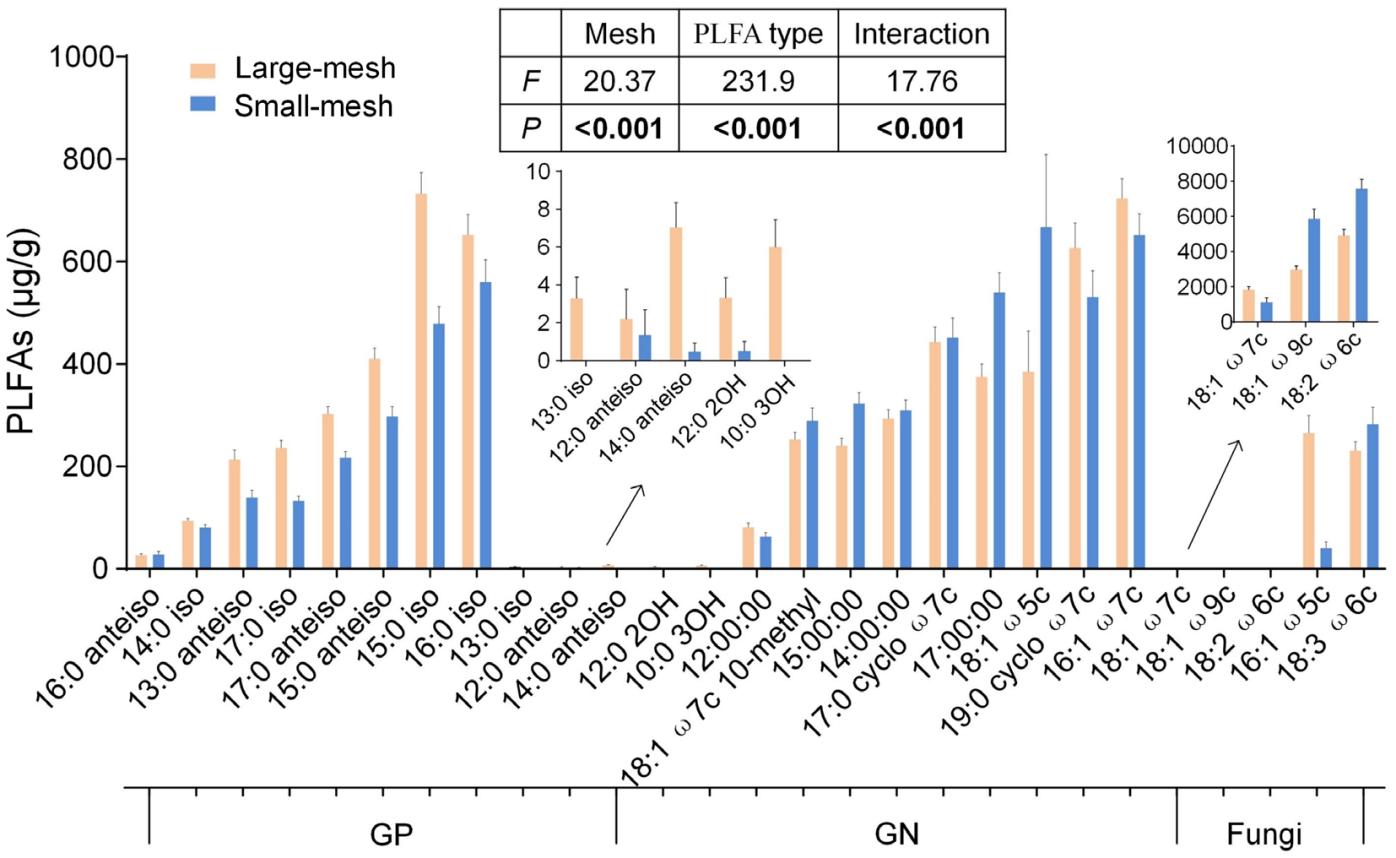
The biomass of phospholipid fatty acids (PLFA) in different litterbag types. Black and gray bars represent PLFA biomass in large‐mesh and small‐mesh litterbags. GN, gram‐negative bacteria; GP, gram‐positive bacteria.

A total of 27 microbial phospholipid fatty acids (PLFA) were covered in this study (Figure 2). The overall fungal PLFA of the 15 litter combinations was significantly higher than that of bacteria (*t* = 7.77, *p* < 0.001), while Gram‐negative bacteria PLFA (GN) were significantly higher than Gram‐positive bacteria (GP, *t* = 14.16, *p* < 0.001). The soil fauna significantly influenced microorganisms, affecting both microbial PLFA and community distribution (Figures 2 and S2).

**FIGURE 3:**
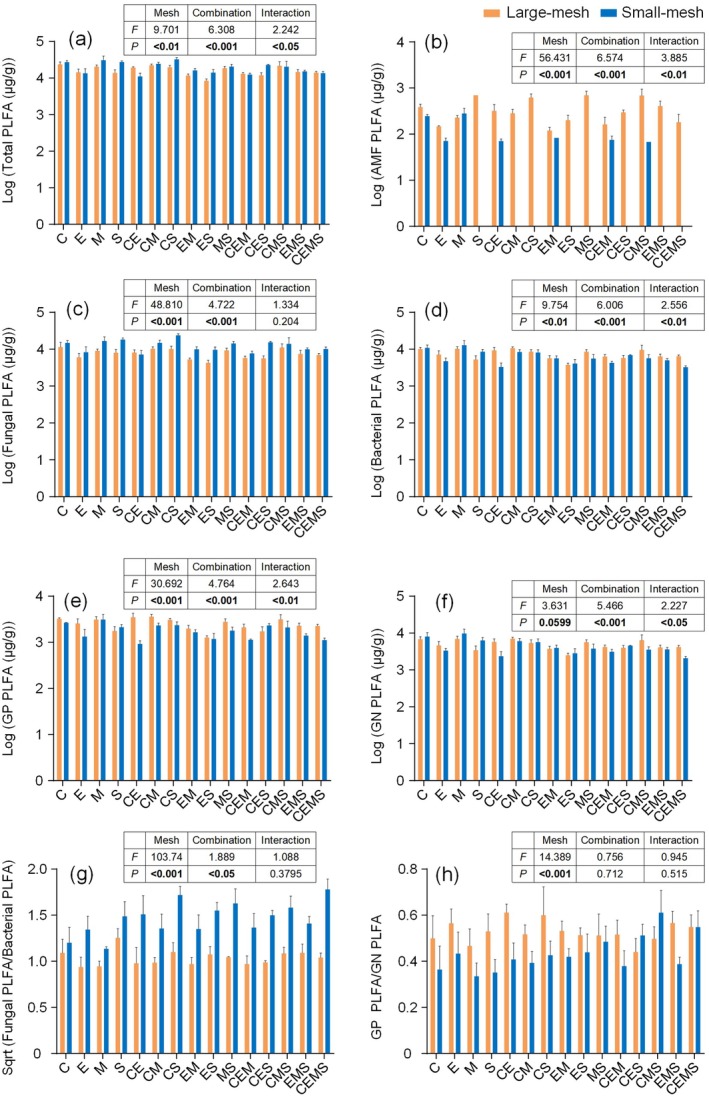
Two‐way analysis of variance for total microbes, AMF, fungus, bacteria, GP, GN, the ratio of Gram‐positive bacteria to Gram‐negative bacteria, and the ratio of fungi to bacteria under different combinations and litterbag types. AMF, arbuscular mycorrhizal fungi; C, *Castanopsis fargesii*, E, *Elaeocarpus japonicus*; GN, Gram‐negative bacteria; GP, Gram‐positive bacteria; M, *Machilus nanmu*, S, *Symplocos lucida*.

In the large‐mesh litterbags, the combinations with the highest mean values of total microbial, fungal, bacterial, GP, and GN PLFA were C, C, CM, CE, and CMS, respectively, whereas the ES had the lowest contents for all of them (Figure 3a–f). The combinations with the highest mean values of F/B and GP/GN were S and CE, respectively, whereas the lowest combinations were E and CES, respectively (Figure 3g,h). Notably, arbuscular mycorrhizal fungi (AMF) were detected in the S, CM, CS, ES, MS, CES, EMS, and CEMS mixtures only when soil fauna had access to the litterbags (Figure 3b).

In the small‐mesh litterbags, the combinations with the highest and lowest mean values of fungal PLFA were CS and CE, respectively (Figure 3c). The combinations with the highest mean values of total microbial, bacterial, GP, and GN PLFA were all M, and the lowest combinations were CE, CEMS, CE, and CEMS, respectively (Figure 3a–f). The combinations with the highest mean values of F/B and GP/GN were CEMS, CMS, and the lowest combinations were all M (Figure 3g,h).

### 3.3 Drivers of Phospholipid Fatty Acids

Total PLFA, PLFA of bacteria and fungi were significantly affected by litter richness, litter combination, and litterbag types (Table 2). Also, bacteria, GP, GN, and AMF PLFA were influenced by the interaction between litterbag type and litter combination (Figure 3).

**TABLE 2:** Permutation multivariate ANOVA test evaluates the effects of litter richness, litter combination, and their interactions on the microbial phospholipid fatty acids (PLFA) based upon different litterbag types.

Source of variation	df	Large‐mesh				Small‐mesh			
		SS	*F*	*R* ^2^	*p*	SS	*F*	*R* ^2^	*p*
PLFA									
Richness	3	0.14	1.10	0.04	0.37	0.72	4.60	0.13	**< 0.001**
Combination	11	1.47	3.16	0.43	**< 0.001**	2.37	4.14	0.44	**< 0.001**
Residuals	43	1.82		0.53		2.29		0.43	
Bacteria									
Richness	3	0.13	1.03	0.04	0.44	0.72	4.48	0.13	**< 0.001**
Combination	11	1.46	3.22	0.43	**< 0.001**	2.37	4.04	0.44	**< 0.001**
Residuals	43	1.77		0.53		2.35		0.43	
Fungi									
Richness	3	0.22	1.39	0.06	0.20	0.67	4.54	0.13	**< 0.001**
Combination	11	1.51	2.60	0.38	**< 0.01**	2.37	4.37	0.45	**< 0.001**
Residuals	43	2.28		0.57		2.17		0.42	

*Note:* Bold indicates significant impact.

**FIGURE 4:**
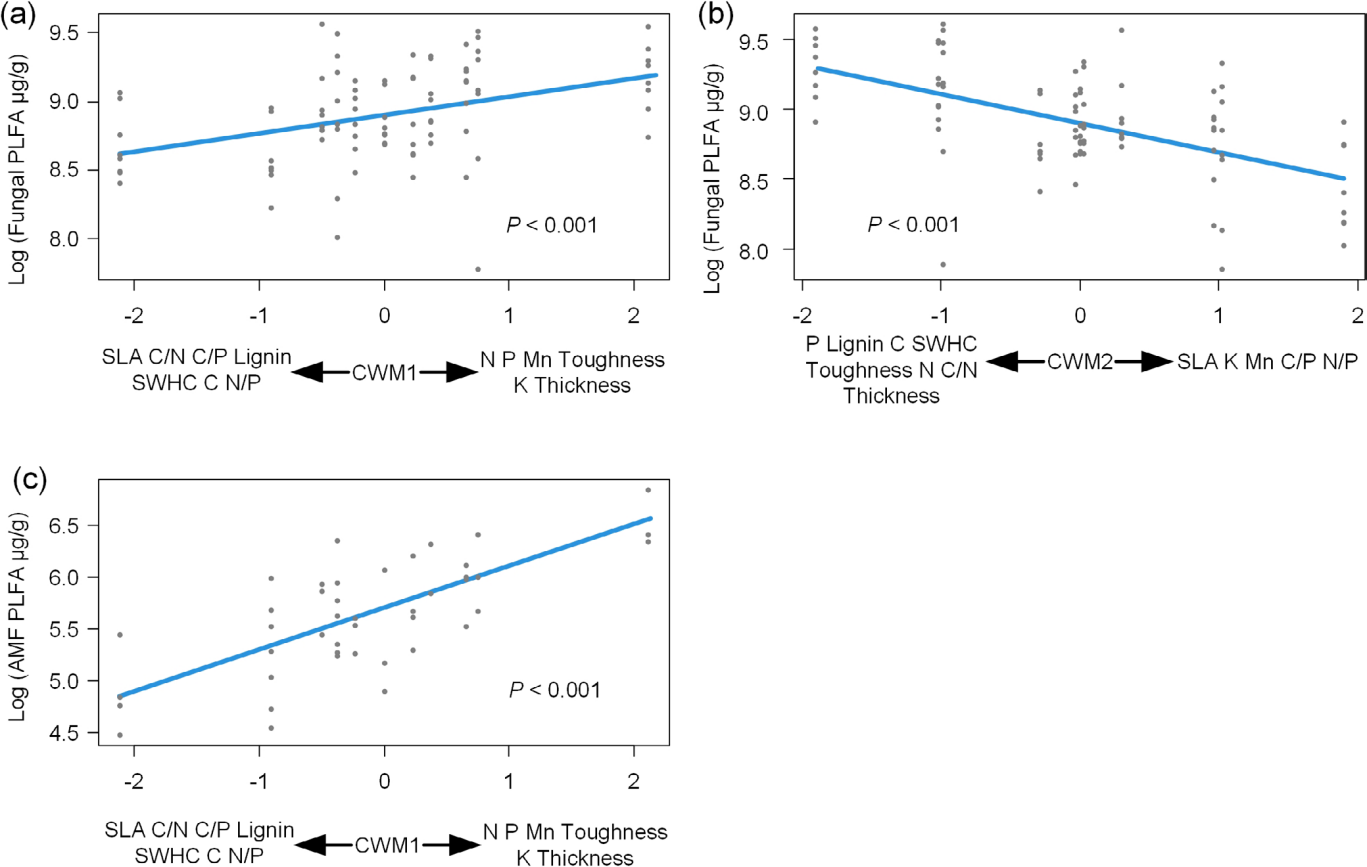
Analyzing the effects of traits on fungal phospholipid fatty acids (PLFA) using linear mixed‐effects models. AMF, arbuscular mycorrhizal fungi; CWM, community‐weighted mean; SWHC, standard water‐holding capacity.

**FIGURE 5:**
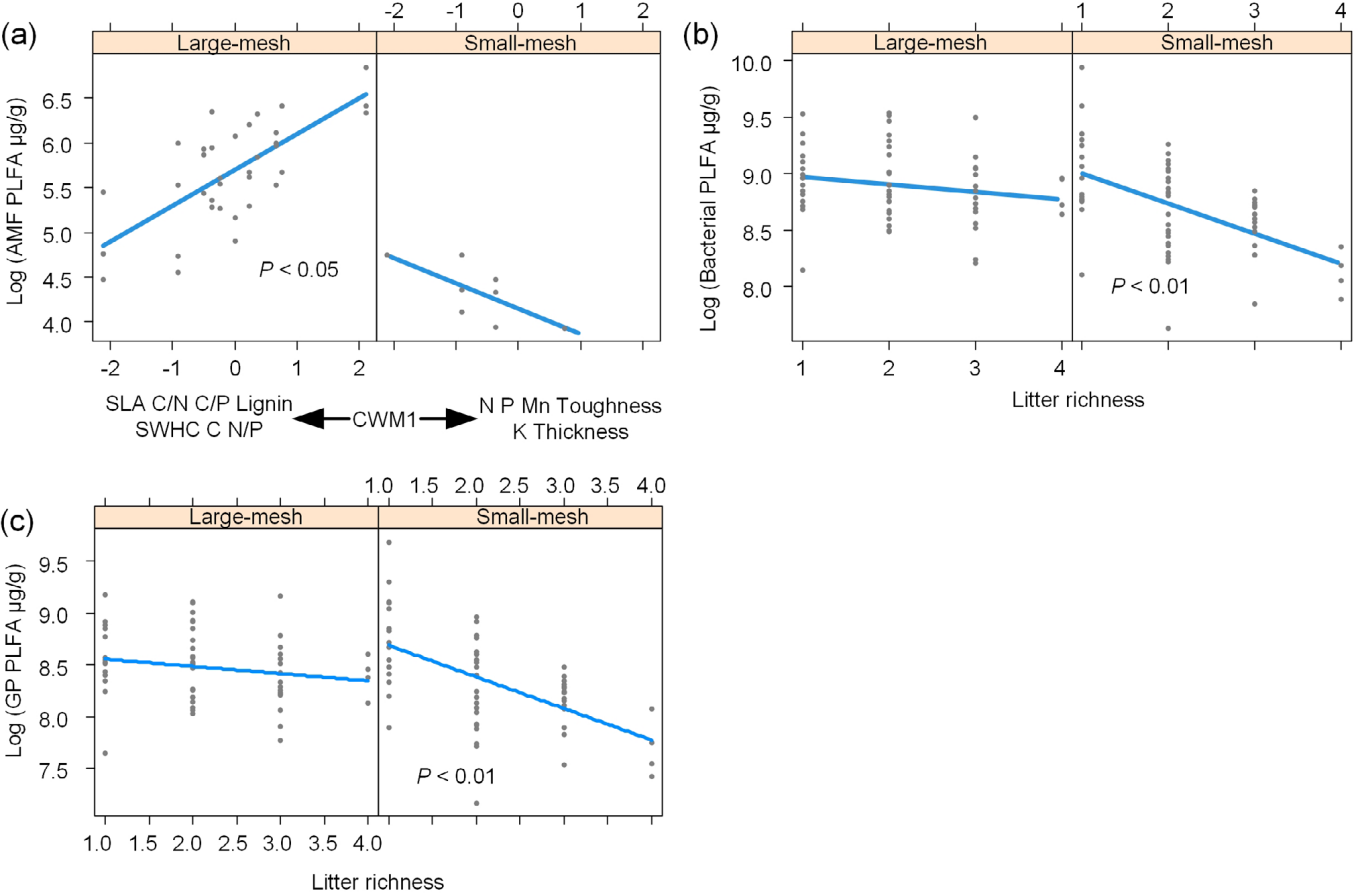
The effects of litter richness and fauna on microbial phospholipid fatty acids (PLFA). AMF, arbuscular mycorrhizal fungi; CWM, community‐weighted mean; GP, Gram‐positive bacteria; SLA, specific leaf area; SWHC, standard water‐holding capacity.

Fungal and AMF PLFA increased with increasing CWM1, i.e., with increasing litter N, P, Mn, toughness, K, and leaf thickness (Figure 4a). However, for AMF, AMF PLFA decreased with increasing CWM1 when soil fauna was removed (Figure 5a).

**FIGURE 6:**
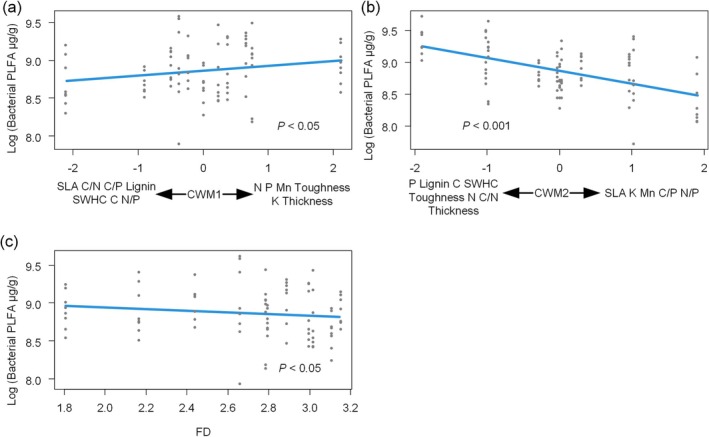
Analyzing the effects of litter trait and richness on bacterial phospholipid fatty acids (PLFA) using linear mixed‐effects models. CWM, community‐weighted mean; FD, functional diversity; SWHC, standard water‐holding capacity.

Bacterial PLFA increased with CWM1 and decreased with FD (Figure 6). Meanwhile, bacterial PLFA decreased with increasing litter richness and was affected by the interaction between litter bag types and litter richness. In other words, the decline in bacterial PLFA with increasing litter richness was steeper when soil fauna was excluded (Figure 5b).

**FIGURE 7:**
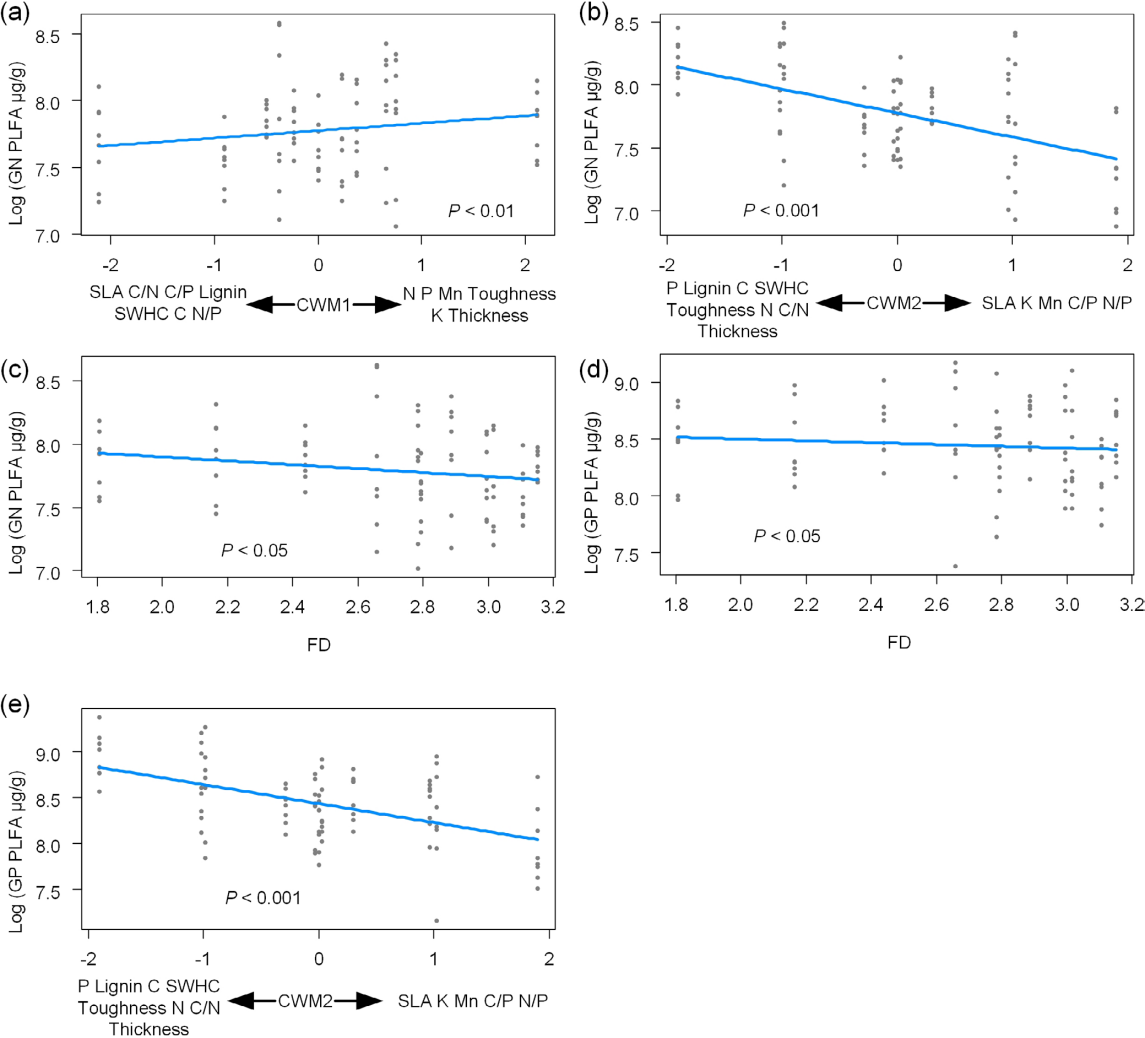
Analyzing the effects of litter trait and richness on Gram‐positive (GP) and Gram‐negative (GN) bacterial phospholipid fatty acids (PLFA) using linear mixed‐effects models. CWM, community‐weighted mean; FD, functional diversity; SWHC, standard water‐holding capacity.

Both GP and GN PLFA decreased with increasing CWM2, FD (Figure 7b–e). Meanwhile, GP PLFA decreased with increasing litter richness and was influenced by the interaction between litterbag types and litter richness. That is, GP PLFA decreased more rapidly with increasing litter richness when soil fauna was removed (Figure 5c).

## 4 Discussion

This study offers a comprehensive analysis of the microbial community structure involved in litter decomposition and the driving mechanisms behind its changes, based on in situ decomposition experiments across various trophic levels. The results indicated that litter traits and soil fauna were strongly linked to microorganisms through distinct pathways, namely bottom‐up and top‐down processes, respectively. Notably, soil fauna played a crucial regulatory role in shaping the interaction between litter mixtures and microorganisms.

The effects of mixed litter traits and richness on microbial PLFA aligned with our first hypothesis. Firstly, total PLFA and all types of bacterial PLFA in *Machilus nanmu* (M) were higher in the absence of animal participation, likely due to its lowest N/P values and highest standardized water holding capacity. The N/P ratio of the litter is a crucial determinant of litter decomposition (Gusewell and Gessner 2009; Song et al. 2020). Previous research consistently shows that litter with N/P ratios greater than 22 tends to exhibit P‐limitation during decomposition (Gusewell and Freeman 2005). This may arise because microorganisms are limited by insufficient P availability. For example, the combination of *Elaeocarpus japonicus* and *Symplocos lucida* (ES), with an N/P ratio of 28.39, exhibited the lowest microbial PLFA across all microorganism types when soil fauna was involved. Meanwhile, when considering the C/P ratio of the mixed litter and its fungal PLFA, we further found that fungi may also be limited by P. For instance, mixed litter with species *Elaeocarpus japonicus* showed a higher C/P ratio but lower fungal PLFA, while mixed litter with species *Symplocos lucida* showed a lower C/P ratio but higher fungal PLFA. Furthermore, the standard water holding capacity of litter plays an important role in maintaining water balance and facilitating the hydrological cycle within forest ecosystems (Li et al. 2013). A previous study found that their standard water‐holding capacity influences the performance of microorganisms (Li et al. 2018). Litter with a higher water storage capacity may improve the leaching process, providing favorable conditions for microorganisms (Schroeter et al. 2022). Additionally, secondary metabolites, which may leach out during this process, could further impact microbial communities by influencing microbial growth and activity (Sana et al. 2025). A consensus has emerged regarding the role of litter traits in shaping microbial community composition (Wan et al. 2022). Second, functional diversity is closely related to microbial PLFA. We did not observe a positive effect of increased functional diversity on decomposer abundance (Xiao et al. 2020). In contrast, bacterial PLFA decreased with increasing functional diversity, including both GP and GN, likely due to the complex trophic cascade effects during litter decomposition (Lenoir et al. 2006; Zhu et al. 2021). Third, litter richness can significantly influence decomposer activity by enhancing resource complementarity and modifying microhabitats (Hattenschwiler et al. 2005; Li et al. 2014; Liu et al. 2022). Additionally, litter richness might create more opportunities for diverse functional traits. Our study revealed that litter richness influenced the biomass of different microbial types and shaped the structure of the microbial community. Specifically, we observed a significant decrease in bacterial biomass with increasing litter richness, which may have been detrimental to the degradation of labile substances in the soil (Paterson et al. 2008).

Soil fauna significantly influenced microbial community structure and PLFA, supporting the second hypothesis. Previous studies have shown that soil animals contribute to the decomposition of forest litter, accounting for approximately 30% of this process (Xu et al. 2020). The feeding niches of soil fauna change constantly as they experience variations in size, metabolism, and physiological functions throughout their lifetime (Fontana et al. 2019). These changes can indirectly impact the decomposition microenvironment (Bradford et al. 2002) and further amplify the differences in traits among litter species. In line with this, the addition of litter significantly increased the total PLFA content of soil microorganisms, fungi, and bacteria, whereas the removal of litter significantly decreased soil animal density but had little impact on the microbial community (Wei et al. 2022). This suggests that soil microbial communities are more sensitive to changes in carbon sources, while the role of soil animals appears to be more indirect, likely influencing microbial community structure through their effect on litter characteristics. Furthermore, soil invertebrates substantially affect the structure of microbial communities through top‐down effects within the food chain (Bradford et al. 2002). In this study, differences in the distribution of microbial species highlighted the role of soil fauna in shaping the microbial community structure. For instance, some combinations, such as S, CM, CS, ES, MS, CES, EMS, and CEMS, involve AMF only in the presence of soil fauna. This finding suggests that distinct microbial communities associated with different soil fauna may contribute to maintaining arbuscular mycorrhizal fungal (AMF) PLFA and its associated benefits (Zhu et al. 2021). AMF are known for their roles in balancing nutrient stoichiometry and regulating microbial metabolism. They alleviate the limitations of microbial carbon and phosphorus by reducing the microbial carbon‐to‐phosphorus ratio (Mei et al. 2022). Despite the reduced proportion of fungi resulting from the evenness and abundance of the soil fauna, the biomass of AMF remained relatively high.

Soil fauna modified the relationship between litter traits and microbial PLFA, supporting our third hypothesis. On one hand, soil fauna appeared to eliminate or modify the effect of litter richness on microbial PLFA. Considering litter's physical and chemical properties, higher litter richness may promote effective niche utilization by the soil biota (Hector et al. 2000). On the other hand, soil fauna significantly affected the relationship between mixed litter traits (CWM) and AMF PLFA, reversing the negative correlation observed in the small‐mesh litterbags to a positive one in the large‐mesh litterbags. Here, soil fauna may enhance resource competition within clumped mycorrhizal fungal communities by preying on other fungi (Crotty and Adl 2019). AMF can acquire substantial amounts of nitrogen from litter, which may contribute to their growth and adaptation to soil fauna (Hodge and Fitter 2010). Additionally, the release of nutrients from recalcitrant materials to support the growth of other microorganisms may underscore the complementarity between arbuscular mycorrhizal fungi and various microbial taxa (Bhatnagar et al. 2018). Although measuring fatty acid content provides valuable information about the microbial community composition, it does not offer detailed insights into specific functional groups or their responses to the biological factors studied. This limitation is particularly relevant when considering how decomposition rates can vary across different studies, which may be attributed to the varying performances of microbial groups at different stages of decomposition (Chen et al. 2021; Wu et al. 2014). By incorporating this temporal dimension, we could differentiate between the contributions of specific microbial groups, such as arbuscular mycorrhizal fungi, at various stages of the process. Therefore, while PLFA is valuable for assessing microbial community composition, its limitation in capturing functional dynamics hinders a full understanding of microbial involvement in decomposition. To address these limitations, we suggest that future studies could incorporate advanced techniques like metagenomics or stable isotope probing (Zhou et al. 2022). These methods could provide a more detailed understanding of the functional diversity and dynamics of microorganisms throughout the decomposition process, complementing the insights gained through PLFA. Furthermore, our study confirmed that relationships between mixed litter characteristics and microbial PLFA were vulnerable to disturbance by soil fauna. However, our design, pertaining to litterbags and sampling time, did not support the investigation of the role of soil fauna of various sizes at different stages of litter decomposition. This limitation includes the study of the distinct microbiomes associated with different functional types of soil fauna and their interactions.

Litter decomposition is a key process in the carbon and nutrient cycles of ecosystems. Even under similar environmental conditions, litter decomposition dynamics can be influenced by multiple factors, particularly interactions among complex biological factors related to biodiversity. In this study, we used microorganisms as a bridge between top‐down and bottom‐up models to explore interactions across biodiversity at different trophic levels. Our study indicated that changes in mixed litter traits, litter richness, and soil fauna are closely connected to microbial activity. Further field studies are needed to examine how their interactions unfold at different stages of decomposition.

## Author Contributions

**Pengpeng Dou:** data curation (equal), formal analysis (lead), funding acquisition (supporting), investigation (equal), methodology (equal), software (lead), writing – original draft (lead). **Dunmei Lin:** conceptualization (lead), data curation (equal), funding acquisition (lead), investigation (equal), methodology (equal), resources (lead), writing – review and editing (lead).

## Funding

This work was supported by the Fundamental Research Funds of CAF (CAFYBB2025QD008), Inner Mongolia Autonomous Region Natural Science Fund (2025QN03199), National Natural Science Foundation of China (31500356).

## Conflicts of Interest

The authors declare no conflicts of interest.

## Supporting information


**Figure S1:** Principal component analysis of CWM trait. SWHC, standard water holding capacity; SLA, specific leaf area; C, *Castanopsis fargesii*, E, *Elaeocarpus japonicus*, M, *Machilus nanmu*, S, *Symplocos lucida*.
**Figure S2:** Nonmetric multidimensional scaling (NMDS) ordination based on Bray–Curtis similarity matrices depicting microbial distribution.

## Data Availability

The datasets generated during and/or analyzed during the current study are available in Dou and Lin (2025a). Dou, P., Lin, D., 2025. The role of soil fauna in modulating the effect of mixed litter diversity on microbial communities during decomposition [Data set]. Zenodo. https://doi.org/10.5281/zenodo.15783843.
