# Supplementary material for: Soil Fauna Alter the Relationship Between Plant Litter Diversity and Microbial Communities in Mixed Litter Decomposition
Source: Ecol Evol. 2026 Jan 30;16(2):e72992. doi: 10.1002/ece3.72992 (PMC12856379; doi:10.1002/ece3.72992)
Supplement: Supplementary file 1 — Figure S1: Principal component analysis of CWM trait. SWHC, standard water holding capacity; SLA, specific leaf area; C, Castanopsis fargesii, E, Elaeocarpus japonicus, M, Machilus nanmu, S, Symplocos lucida. Figure S2: Nonmetric multidimensional scaling (NMDS) ordination based on Bray–Curtis similarity matrices depicting microbial distribution. [file ECE3-16-e72992-s001.docx]

**Soil fauna alter the relationship between plant litter diversity and microbial communities in mixed litter decomposition**

This file contains:

Supplementary Figures S1-S2


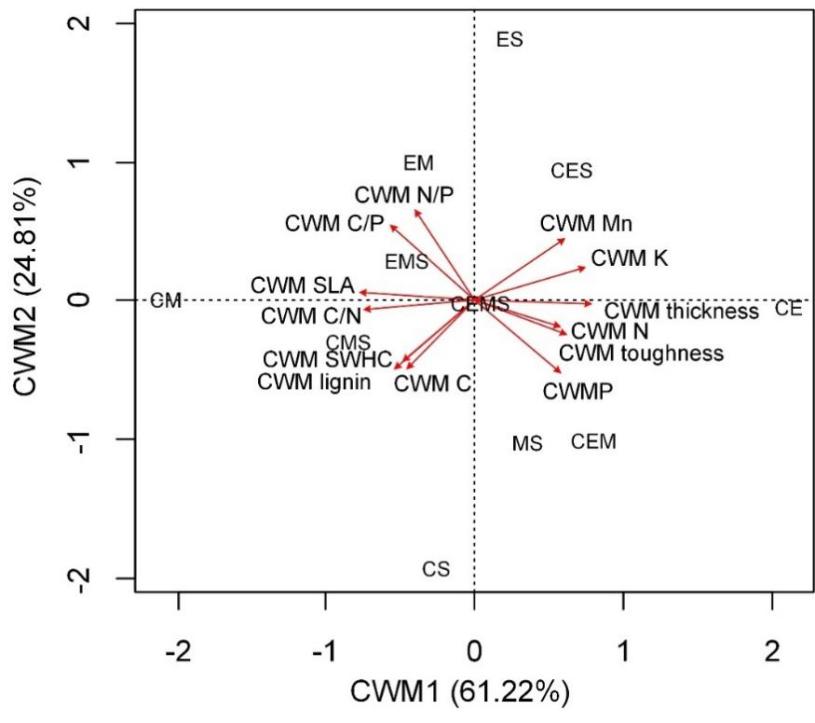


Fig. S1 Principal component analysis of CWM trait. SWHC, standard water holding capacity; SLA, specific leaf area; C, *Castanopsis fargesii*, E, *Elaeocarpus japonicus*, M, *Machilus nanmu*, S, *Symplocos lucida*.


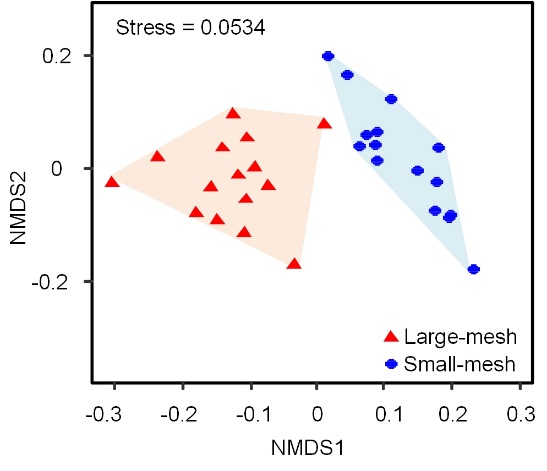


Fig. S2 Nonmetric multidimensional scaling (NMDS) ordination based on Bray–Curtis similarity matrices depicting microbial distribution.
